# Seroprevalence of hepatitis B virus among hemodialysis patients in Bushehr province, southern Iran: HBV seroprevalence in hemodialysis patients

**Published:** 2011-03-01

**Authors:** Amir Ahmad Mostaghni, Alireza Soltanian, Elaheh Mokhtari, Sara Japoni, Davood Mehrabani

**Affiliations:** 1 Gastroenterohepatology Research Center, Nemazee Hospital,Shiraz University of Medical Sciences, Shiraz, IR Iran; 2 Department of Internal Medicine, Bushehr University of Medical Sciences, Bushehr, IR Iran; 3 Stem Cell and Transgenic Technology Research Center,Shiraz University of Medical Sciences, Shiraz, IR Iran

**Keywords:** Hepatitis B virus, Anti-HBs antibody, Hemodialysis, Prevalence, Iran

## Abstract

**Background:**

Hepatitis B virus (HBV) infection is still reported from adult hemodialysis units.

**Objectives:**

To determine the prevalence of anti-HBs antibody in hemodialysis patients and the correlation between levels of anti-HBs antibody with other factors.

**Patients and Methods:**

HBsAg, anti-HBs and anti-HBc antibodies level in 119 hemodialysis patients were evaluated by enzyme-linked immunosorbent assay.

**Results:**

Seroconversion (anti-HBs antibody >10 IU/L) was found in 22 patients. Minimum protective antibody level was found in patients aged ≥60 years. Statistically significant correlation was not found between anti-HBs antibody and gender. Ten (8.4%) patients had abnormal ALT and/or AST. Prevalence of HBsAg, anti-HBc antibody, HBeAg and anti-HBe antibody were found in 8 (6.72%), 24 (25.16%), 2 (1.68%) and 3 (2.52%) patients, respectively.

**Conclusions:**

Periodic assessment of anti-HBs antibody level is strongly recommended in patients undergoing hemodialysis.

## Background

Hepatitis B virus (HBV) is a DNA virus transmitted percutaneously, sexually or prenatally affecting 350-400 million persons worldwide. HBV infection globally accounts for one million deaths annually from liver failure, cirrhosis, and hepatocellular carcinoma [1][2][3][4][5]. Compared with the general population, dialysis patients are at high risk of acquiring HBV that may be directly exposed to blood products, shared hemodialysis (HD) devices and breaching of the skin. HD which needs access to the blood circulation, also may lead to transmission of HBV between patients, and between patients and staffs [6]. Viral proteins of clinical importance include the envelope protein, hepatitis B surface antigen (HBsAg), hepatitis B core antigen (HBcAg) and hepatitis B e antigen (HBeAg). Serum HBsAg is considered as a marker of chronic HBV infection [7]. The detection of anti-HBc and anti-HBs antibodies denotes a previous infection [8]. Anti-HBs antibody levels of > 10 IU/L indicate the immunity. There is an inverse correlation between the risk of HBV infection after exposure and the maximal anti-HBs response to vaccination, [9] with an anti-HBs antibody titer of > 100 IU/L conferring very effective protection [10][11].

## Objectives

Due to irregular vaccination of hemodialysis patients in Bushehr province, southern Iran, at the time of the study, this study was carried out to determine the prevalence of HBsAg, anti-HBs antibody, anti-HBc antibody, HBeAg and anti-HBe antibody in HD patients in Bushehr province, southern Iran. Correlations between level of anti-HBs antibody with age, gender, diabetes mellitus (DM), duration of dialysis and fluctuation of liver function tests (AST and ALT) were also assessed in this study.

## Patients and Methods

From September 2004 to September 2005, 119 patients undergoing HD in Bushehr University of Medical Sciences affiliated hospitals were screened for HBsAg, anti-HBs and anti-HBc antibodies. A questionnaire was used to collect demographic data, date of onset of HD, length of HD services and other probable risk factors. History of vaccination against HBV was recorded from patient's medical records. Enzyme-linked immunosorbent assays (ELISA, Biokit, Spain) was used to measure HBsAg, anti-HBs and anti-H anti-HBs antibody Bc antibodies titers. ELISA reader (Thermolab system, Finland) was used to measure the titer of antibody at 450 nm. The HBsAg-positive samples were then sent to Iranian Blood Transfusion Center in Bushehr, southern Iran to be screened for HBeAg and anti-HBe antibody using ELISA (Diasorin, Italy). The data were analyzed by SPSS® for Windows® (ver 15, Chicago, IL, USA). A p value < 0.05 was considered statistically significant.

## Result

A total of 119 HD patients were assessed for HBV serological markers of which 44 (37%) were female and 75 (63%) were male. Correlation between anti-HBs antibody titer and gender was not statistically significant (p > 0.05). The mean ± SD age of patients was 48 ± 14 (range: 10-83) years. The patients were classified into six groups based on their age and anti-HBs antibody titer with a cutoff value of 10 IU/L. The prevalence of protective antibody titer in two age groups including 60-69 and ≥ 70 years were at the lowest level (p = 0.031). From the 119 HD patients, 11f2 (94.1%) had received at least one dose of vaccination. The frequencies of patients who had received one, two and three doses of vaccine were 78 (69.6%), 19 (17%) and 15 (13.4%), respectively. The prevalence of anti-HBs antibody > 10 IU/L and anti-HBs Ab > 100 IU/L were 22 (18.5%) and 7 (5.9%), respecftively. No anti-HBs antibody-positive patients was found positive for anti-HBc antibody. Therefore, the positive anti-HBs antibody was indeed reflected immunity due to vaccination. An increase in the number of vaccination dose, resulted into a rise in the titer of the corresponding antibody ( 1).

**Table 1:** Prevalence of anti-HBs antibody ≥10 IU/L and anti-HBs antibody ≥100 IU/L in different doses of vaccination in 112 hemodialysis patients who had received at least one dose of vaccine

**Number of doses received**	**No **(%)	**Anti-HBs antibody ≥ 10 IU/L **(%)	**Anti-HBs antibody ≥ 100 IU/L **(%)
**1**	78 (69.6)	8 (10)	1 (1)
**2**	19 (17)	5 (26)	3 (16)
**3**	15 (13.4)	8 (53)	3 (20)

The prevalence of anti-HBs antibody ≥ 10 IU/L and anti-HBs antibody ≥ 100 IU/L in different groups of dialysis patients is shown in Table 2. Thirty-two patients had received a complete vaccine dose (3 doses) with long period of dialysis (≥ 4 years). Titer of antibody in 32% of these patients rose to ≥ 10 IU/L; the rate for those who had received only one dose of vaccine was 8.3% (Table 2). Ten (8.4%) patients had abnormal ALT (> 31 U/L in women and > 41 U/L in men) and/or AST (> 31 U/L in women and > 37 U/L in men). Co-incidence of abnormal ALT and/or AST with anti-HBs and anti-HBc antibodies were observed in 3 (2.5%) and 4 (3.3%) patients, respectively. The prevalence of HBsAg, anti-HBc antibody, HBeAg and anti-HBe antibody were 6.72% (No. = 8), 25.16% (No. = 24), 1.68% (No. = 2) and 2.52% (No. = 3), respectively.

**Table 2:** Prevalence of anti-HBs antibody ≥10 IU/L and anti-HBs antibody ≥100 IU/L in different groups of dialysis in 112 patients who had received at least one dose of vaccine

	**Number of doses received **(%)			**≥ 10 IU/L **(%)	**≥ 100 IU/L **(%)	**Total**
**1**	**2**	**3**			
**≤ 1 year**	40 (90)	4 (10)	0 (0)	4 (8)	0 (0)	44 (39.3)
**2 years**	22 (71)	3 (10)	6 (19)	7 (23)	2 (7)	31 (27.7)
**3 years**	5 (42)	6 (50)	1 (8)	3 (20)	1 (7)	12 (10.7)
**≥ 4 years**	11 (44)	6 (24)	8 (32)	8 (32)	2 (8)	25 (22.3)

## Discussion

An increased risk of exposure to HBV infections is observed in patients on chronic HD [12]. It has been shown that after vaccination, dialysis patients develop lower antibody titers compared to healthy individuals, and are unable to maintain adequate antibody titers over time [13]. Distribution of gender in HD patients in the current study was similar to that in local and international reports [14][15][16][17]. Despite a slight higher frequency in male, no association was observed with gender in this study. Old HD patients had routinely a poorer immune response as observed in this study [18][19][20][21]. Lower antibody production in elderly to vaccines could be due to their diminished immune response [22]. The present study was in keeping with other reports that there was not a significant association between presence of DM in HD patients and poor immune response [23][24]. In the present study, the frequency of people with anti-HBs antibody > 10 IU/L was lower than that reported in other studies from Iran and other countries [14][15][16][17][25][26][27][28]. This can be explained by the fact that only 13.4% of HD patients studied had received a complete vaccination schedule, hence, antibody titer has not reached a protective level. Besides, lack of information on the date of previous vaccination or response to vaccines can interfere with accurate interpretation of the results. In this investigation, the prevalence of HBsAg was 6.72%. National reports show a lower prevalence rate of 3.7% in Semnan, 1.7% in Kashan [29], 2.4% in Tehran in 2005 [16], and 2.4% in 2008 [29]; international reports indicate a prevalence ranging from 5.88 % in Bahrain and Saudi Arabia [30] to 15.4% in Brazil [31]. In a study by Alavian et al. the prevalence of HBsAg has been decreased from 3.8% in 1999 to 2.6% in 2006 among HD patients in Iran [32]. Similar to our findings (%25.16%), anti-HBc antibody was shown to be 21.5% in southeastern Iran [33]. Ferriera et al. noticed that patients on maintenance HD for more than three years had a 2.6 (95% CI: 1.7-4.0) times higher risk of acquiring HBV infection compared to those who had undergone HD for less than 12 months [34]. This correlation was not found to be significant for HBV infection in the present study. There is a low prevalence rate of positive anti-HBs antibody in HD units. Therefore, HD patients should take a complete vaccination schedule and be monitored afterwards. In case of inadequate antibody titer, these patients should receive an additional dose of vaccine.
